# Management of chest keloids

**DOI:** 10.1186/1749-8090-6-49

**Published:** 2011-04-13

**Authors:** Tae Hwan Park, Sang Won Seo, June Kyu Kim, Choong Hyun Chang

**Affiliations:** 1 Department of Plastic and Reconstructive Surgery, Kangbuk Samsung Hospital, Sungkyunkwan University School of Medicine, Seoul, Korea

## Abstract

Keloid formation is one of the most challenging clinical problems in wound healing. With increasing frequency of open heart surgery, chest keloid formations are not infrequent in the clinical practice. The numerous treatment methods including surgical excision, intralesional steroid injection, radiation therapy, laser therapy, silicone gel sheeting, and pressure therapy underscore how little is understood about keloids. Keloids have a tendency to recur after surgical excision as a single treatment. Stretching tension is clearly associated with keloid generation, as keloids tend to occur on high tension sites such as chest region. The authors treated 58 chest keloid patients with surgical excision followed by intraoperative and postoperative intralesional steroid injection. Even with minor complications and recurrences, our protocol results in excellent outcomes in cases of chest keloids.

## Background

Keloids are relatively resistant to treatment, with high recurrence rates using a single treatment modality. Keloids have a tendency to recur after surgical excision as a single treatment, with rates approximately up to 80-100%. Keloids can arise from skin trauma and must be removed through skin truma. Therein lies the challenge of treatment, where recurrence would seem inevitable. Surgical excision is considered as a kind of skin trauma and it promotes additional collagen synthesis, resulting in regrowth and even larger keloids[1]. This is why we were focused on the article recently published in your esteemed journal by Patel et al.[2] that dealt with the challenging topic of chest keloids.

## Patients and Methods

58 patients were treated with surgical excision combined with intraoperative/postoperative intralesional steroid injection therapy over a period of six years from July 2003 to June 2009 at our hospital. In all patients, a follow-up period of 18 months was required. Treatment outcome was assessed with global aesthetic improvement score (GAIS). All statistical analyses were conducted using SPSS version 17.0 (SPSS, Inc., Chicago, IL, USA). Our data were not normally distributed; consequently non-parametric tests were used. Descriptive statistics are presented as medians with interquartile ranges or as numbers and percentages.

## Results

**Figure 1:**
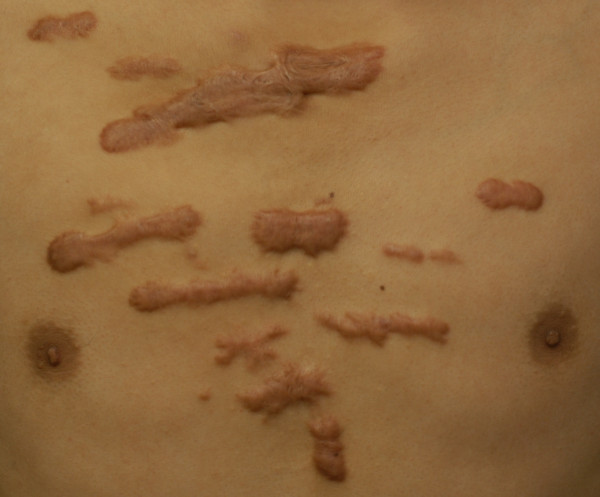
**Chest keloid after acne scar**.

**Figure 2:**
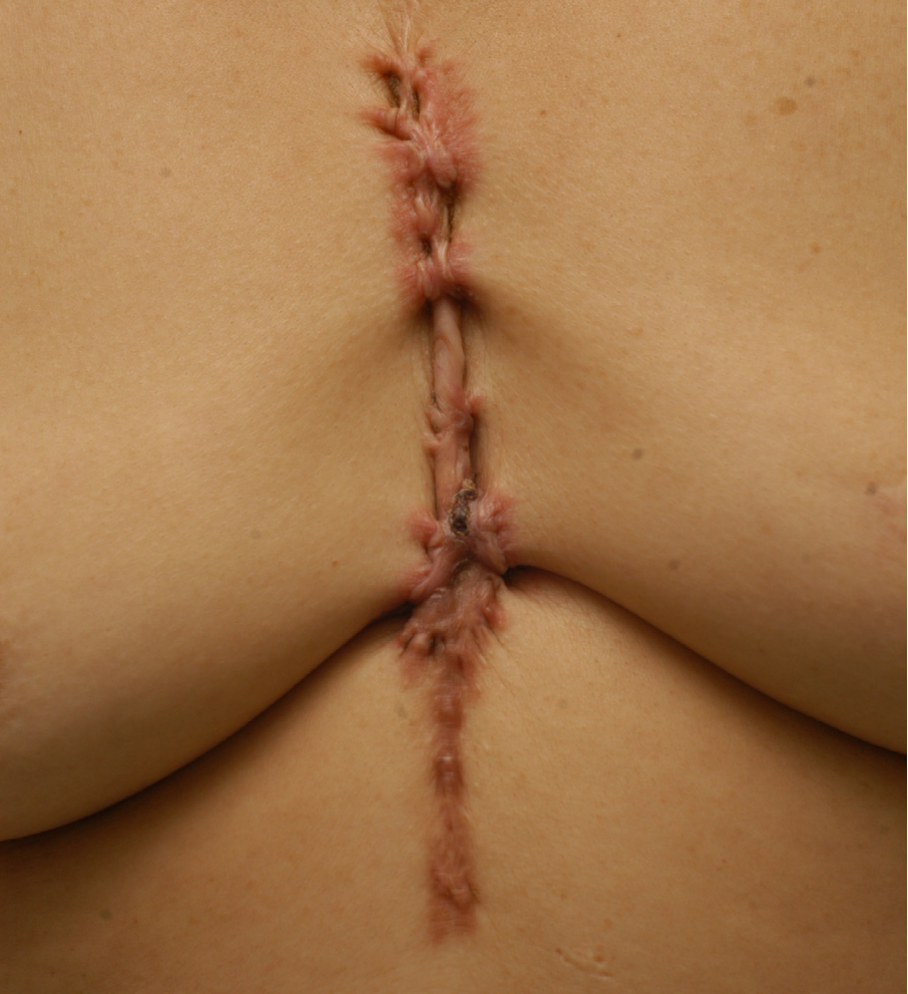
**Chest keloid after open heart surgery**.

**Figure 3:**
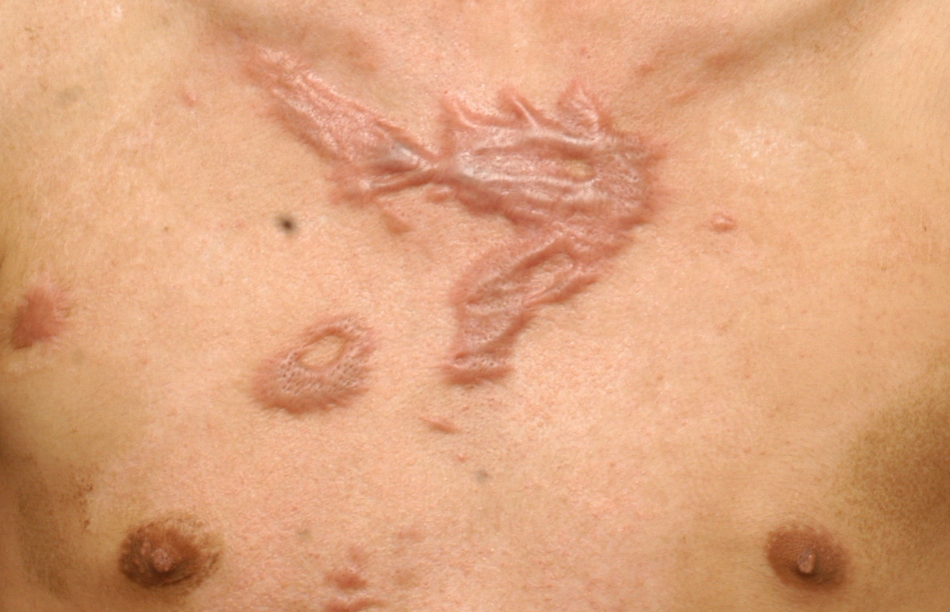
**Chest keloid after severe burn injury**.

**Figure 4:**
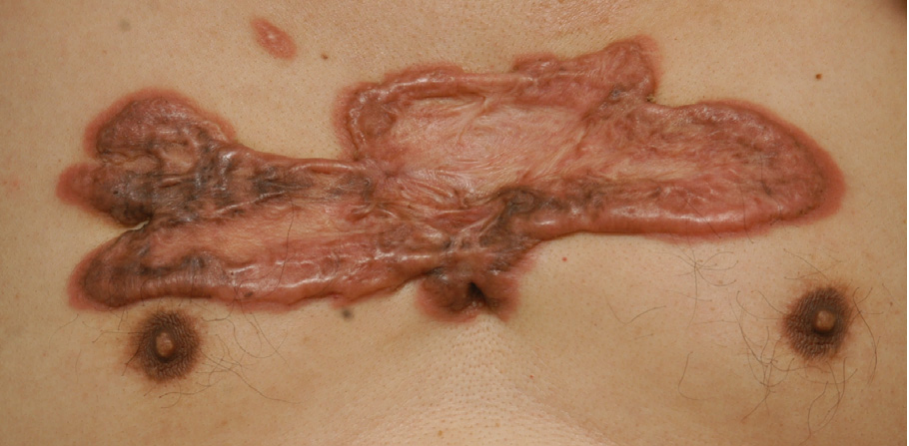
**Chest keloid after shell splinters injury**.

41 (70.7%) were women and 17 (29.3%) were men. The average age was 32 (range 29-35). The average time interval between keloid formation (or prior complete treatment) and time of treatment was 6 (range 5-7) years. The average pretreatment total size of lesions was 3.5 (range 2.0-5.0). 45 patients (29.3%) were treated for a treatment-resistant keloid that failed to respond to previous interventions. These included surgical excision (2 patients, 3.4%), intralesional steroid injection (33 patients, 56.9%), laser therapy (5 patients, 8.6%), acupuncture (3 patients, 5.3%), and cryotherapy (2 patients, 3.4%). The etiologies of chest keloid, in order of decreasing frequency, were the acne scar (20 patients, 34.5%, Figure 1), cardiothoracic surgery (12 patients, 20.7%; Figure 2), burn scar (10 patients, 17.2%; Figure 3), infection (10 patients, 17.2%) and trauma (6 patients, 10.4%; Figure 4). (Table 1)

**Table 1:** Baseline Patient Characteristics

	Total Patients (n = 58)
Age, years	32.00 (29.00-35.00)
Total size, cm	3.50 (2.00-5.00)
Age of keloids, years	6.00 (5.00-7.00)
BMI, kg/m^2^	23.00 (21.00-25.00)
Gender:	
Female, n (%)	41 (70.7%)
Male, n (%)	17 (29.3%)
Previous treatment history:	
No, n (%)	13 (22.4%)
Yes, n (%)	45 (77.6%)
Surgical excision, n (%)	2 (3.4%)
Steroid injection, n (%)	33 (56.9%)
Laser therapy, n (%)	5 (8.6%)
Acupuncture, n (%)	3 (5.3%)
cryotherapy, n (%)	2 (3.4%)
Etiology:	
Acne scar, n (%)	20 (34.5%)
Cardiothoracic surgery, n (%)	12 (20.7%)
Burn scar, n (%)	10 (17.2%)
Infection, n (%)	10 (17.2%)
Idiopathic, n (%)	6 (10.4%)

Values are median(IQR) for continuous variables and number (percentages) for categorical variables.

## Discussion

Although various surgical techniques are introduced in the medical literature, surgical excision alone is inadequate considering high recurrence rate of keloids[3]. In the cases of chest keloids, our treatment protocol was surgical excision with intraoperative and postoperative intralesional steroid injections. Patients were informed of the possible keloid recurrence and were told to return if a scar was reelevated or extended beyond the demensions of the initial lesion. Even with minor complaints, such as pruritus, pain, tenderness, and secondary infection, most patients were satisfied with the outcomes. Diverse adjuvant methods after surgical excision including intralesional corticosteroids injection, pressure therapy, radiation therapy, topical silicone-gel sheeting, cryotherapy, and laser treatment have been proposed for keloids. In the chest keloids, radiation therapy cannot be the primary adjuvant therapy because of its possible risk of radiation-induced malignancy. Thyroid and breast carcinoma after radiation therapy for keloids have been reported in the medical literatures[4]. In addition, various pressure devices cannot be properly applied on the chest region[5,6]. Even though silicone gel is comfortable and sometimes useful, it requires active patient compliance and long-term application can be challenging[7].

We also stress adequate follow-up periods are mandatory to properly assess the outcome of treatment protocol. According to available literatures, at least 12 months follow period is recommended.

## Conclusions

Although the exact pathogenesis of keloid remains unclear, stretching tension is clearly associated with keloid generation, as keloids tend to occur on high tension sites such as chest region. Therefore, it is difficult to completely eradicate keloids from this region. Even with minor complications and recurrences, we think surgical excision with intraoperative and postoperative intralesional steroid injection remains the treatment of choice in the chest keloids.

## Informed consent

Written informed consent was obtained from the patient for publication of this article and accompanying images. A copy of the written consent is available for review by the Editor-in-Chief of this journal.

## Competing interests

The authors declare that they have no competing interests.

## Authors' contributions

TH was responsible for the conception and design for the manuscript, the clinical work, the search for the literature, and the editing work. JK helped in the clinical work as well as the design for the manuscript. SW edited the manuscript and helped on the clinical work. CH provided overall supervision and contributed to concept. All authors read and approved the final manuscript.
